# Insights into the Role and Interdependence of Oxidative Stress and Inflammation in Liver Diseases

**DOI:** 10.1155/2016/4234061

**Published:** 2016-12-14

**Authors:** Sha Li, Ming Hong, Hor-Yue Tan, Ning Wang, Yibin Feng

**Affiliations:** School of Chinese Medicine, Li Ka Shing Faculty of Medicine, The University of Hong Kong, Pokfulam, Hong Kong

## Abstract

The crucial roles of oxidative stress and inflammation in the development of hepatic diseases have been unraveled and emphasized for decades. From steatosis to fibrosis, cirrhosis and liver cancer, hepatic oxidative stress, and inflammation are sustained and participated in this pathological progressive process. Notably, increasing evidences showed that oxidative stress and inflammation are tightly related, which are regarded as essential partners that present simultaneously and interact with each other in various pathological conditions, creating a vicious cycle to aggravate the hepatic diseases. Clarifying the interaction of oxidative stress and inflammation is of great importance to provide new directions and targets for developing therapeutic intervention. Herein, this review is concerned with the regulation and interdependence of oxidative stress and inflammation in a variety of liver diseases. In addition to classical mediators and signaling, particular emphasis is placed upon immune suppression, a potential linkage of oxidative stress and inflammation, to provide new inspiration for the treatment of liver diseases. Furthermore, since antioxidation and anti-inflammation have been extensively attempted as the strategies for treatment of liver diseases, the application of herbal medicines and their derived compounds that protect liver from injury via regulating oxidative stress and inflammation collectively were reviewed and discussed.

## 1. Introduction

Liver disease, a broad spectrum of disease ranging from early steatosis to severe hepatitis, fibrosis, cirrhosis, and hepatocellular carcinoma (HCC), has high prevalence worldwide. Hepatic diseases could be triggered by various risk factors including obesity, virus, alcohol, drugs, and other toxins [1]. As liver is a central organ detoxification and nutrients metabolism, it is more vulnerable to oxidative stress and inflammation produced from toxins and metabolites in the body [2]. A huge number of animals studies and clinical trials have demonstrated that sustained oxidative stress and inflammation in the liver are crucial in the initiation and development of hepatic illness, regardless of the etiology [3]. Both of them are considered to be the key elements in the pathogenesis of acute and chronic liver diseases. Oxidative stress causes hepatic damage by provoking alteration of biological molecules such as DNA, proteins, and lipids and, notably, modulating biological pathways associated with genes transcription, protein expression, cell apoptosis, and hepatic stellate cell activation [2]. Regarding inflammation, it is an essential component of the immune response and manifested as infiltration of inflammatory cells to primarily liver for fighting against pathogens invasion; however, once the stimuli exist persistently or overwhelmingly, they in turn lead to cellular injury and lipid accumulation associated with increased risk of severe liver diseases such as steatohepatitis, fibrosis, and cancer [4–6].

Of great interest, investigations focusing on the relationship and interaction of oxidative stress and inflammation have attracted great attention as accumulated evidences indicated that they are tightly correlated and orchestrated to drive the pathophysiological procedure of liver diseases [7–9]. At the early stage of liver diseases, either of them may solely present, but both should participate in the pathogenesis of various liver diseases together at later stages. Besides, a number of reactive oxygen species (ROS) or reactive nitrogen species (RNS) can augment proinflammatory gene expression by provoking intracellular signaling cascade. On the other hand, inflammatory cells could produce more ROS/RNS, resulting in exaggerated oxidative stress at inflammatory lesion [7, 10]. The close interplay of oxidative stress and inflammation thus creates a vicious cycle that promotes the pathogenesis of liver diseases. In general, antioxidative therapy showed unsatisfied outcomes in many clinical trials, though numerous studies demonstrated actual involvement of oxidative stress in the pathogenesis in the disease. This has been called as the phenomenon of antioxidant paradox in medical science [11, 12]. Some researchers proposed that it is the complex and tight related interdependence between oxidative stress and inflammation that are responsible for the failure of antioxidant treatment [7]. For example, antioxidant that only improves oxidative stress pathways but aggravates inflammatory cascades is more likely to get failure in treating liver diseases. Therefore, clarifying the interaction of oxidative stress and inflammation is of great importance for the selection of antioxidants that block oxidative and inflammatory pathways simultaneously. In this review, we will briefly summarize the roles of oxidative stress and inflammation in highly prevalent liver diseases. Then, emphasis will be put upon the discussion on the relationship and interdependence of oxidative stress and inflammation in liver diseases including alcoholic liver disease (ALD), nonalcoholic fatty liver disease (NAFLD), fibrosis, and HCC. More importantly, as anti-inflammatory immune suppressor cells also show linkage with oxidative stress [13], based on data as far now, potential mechanism by which immune suppression mediated by oxidative stress regulating inflammation is proposed herein might offer new inspiration for the treatment of liver disease. Furthermore, many herbal medicines have displayed antioxidant and anti-inflammatory activity for treatment of liver diseases by a vast body of studies [8]. Herbal medicines or purified compounds such as berberine (BBR), curcumin, lipoic acid (LA), and epigallocatechin gallate (EGCG) which protect liver from injury via regulating oxidative stress and inflammation collectively will be briefly summarized and discussed in this review.

## 2. Oxidative Stress and Inflammation in Hepatic Lesion

### 2.1. Targets and Participation of Oxidative Stress in Liver Diseases

Oxidative stress is defined as the imbalance between productions of ROS/RNS generated in the aerobic metabolism and their elimination by antioxidant defense including enzymes such as superoxide dismutases (SOD), catalase (CAT), and glutathione peroxidase (GSH-Px) and nonenzymes particles of electron receptors such as glutathione (GSH) and vitamin C/E, which could be triggered in the liver by diverse factors such as obesity, virus, drugs, alcohol, and other toxins [2]. Under sustained exposure to the above factors, ROS/RNS are generated overwhelmingly, thus leading to substantial damage to cell structure and functions. Targets of these reactive species that cause cellular and tissue injury include lipids, DNA, proteins, and related signaling pathways. Free radicals react with lipids to produce hydroperoxides and endoperoxides, which may suffer fragmentation to generate reactive intermediates, such as malondialdehyde (MDA) and 4-hydroxynonenal (4-HNE), to cause irretrievably covalent adducts with proteins, DNA, and phospholipids, resulting in cell death [14]. Reactive stress induced DNA damage primarily occurs in the mitochondrial genome because (i) mitochondrion is open and circular without histone protection and (ii) close to reactive species that is generated heavily inside mitochondrion compared to nuclear region [15]. Cysteine with the thiol group is a reactive amino acid of proteins in cells, and the formation of disulfide by oxidizing two thiols can result in the alteration of protein function. By targeting thiols of proteins, ROS can alter signaling pathways such as cellular kinases, phosphatases, and transcription factors, which have insightful impacts in cell proliferation, differentiation, and apoptosis leading to hepatocytes injury by stimulating oxidant-induced hepatocyte apoptosis or by restraining cell survival signaling cascades [14]. Cellular kinases, particularly in the mitogen-activated protein kinase (MAPK) family such as extracellular signal-regulated kinase 1/2 (ERK1/2), c-Jun N-terminal kinase (JNK), and p38 MAPK, have a critical role in transducing a multitude of extracellular stimuli by phosphorylating and activating downstream transcription factors. Modulation of protein expression in response to oxidative stress occurs mainly via the activation of redox-sensitive transcription factors such as nuclear factor *κ*B (NF-*κ*B), activator protein-1 (AP-1), early growth response protein 1 (EGR-1), and G proteins [15, 16]. It is worth noting that the fate of hepatocytes mainly depends on intensity and duration of the stimuli, which determines the degree and duration of the activation/inactivation of these redox-sensitive cascades, especially the relative level of activation of NF-*κ*B, ERK1/2, and JNK.

Involvement of oxidative stress in liver diseases has been extensively explored and its crucial impact on the pathogenesis of a variety of liver diseases such as ALD, NAFLD, fibrosis, and HCC has been revealed. In ALD, during the metabolism process of alcohol in the liver, reactive intermediate acetaldehyde, which can react with DNA and proteins to form adducts, causes tissue injury. Activated cytochrome P450 2E1 (CYP2E1) responsible for alcohol breakdown in the case of chronic alcohol exposure commits the generation of ROS, resulting in fatty acids deposition and the progress of hepatic steatosis [17, 18]. Additionally, defense system to remove reactive species is also altered by alcohol, such as peroxisome proliferator activated receptor gamma- (PPAR-*γ*-) coactivator 1 *α*, which can induce activation of various ROS-mediated detoxifying enzymes [19–22]. ROS may lead to excessive alcoholic liver fibrosis and cirrhosis via rebuilding of stellate cells and the extracellular matrix within the liver. With regard to NAFLD, substantial hepatic ROS is produced by excessive angiotensin II and activated CYP2E1, ultimately leading to impaired beta-oxidation and fatty liver [23]. Fibrosis, a wound-healing response to hepatocytes injury, is caused by the overproduction of collagen I. The elevated oxidative stress contributes to fibrogenesis via provoking generation of collagen from activated hepatic stellate cells and release of other profibrogenic cytokines, growth factors, and prostaglandins [24–26]. Concerning liver cancer, ROS generated by hypoxia along the edge of tumor growth presents a dual role: (i) it promotes carcinogenesis by activating NF-*κ*B and HIF1*α*, which favor cancer cell survival, angiogenesis, and tumor expansion; (ii) on the other hand, after mitochondrial GSH depletion, it shifts hypoxia from a cancer-promoting to a cancer-killing environment [27–29]. It is proposed that the role of oxidative stress in cancer depends on the time course: destructive role in the early phase of carcinogenesis and protective role in late phase.

### 2.2. Participation of Inflammation in Liver Diseases Spectrum

When the liver is challenged by exogenous and endogenous stimuli like virus, allergens, toxins, or obesity, inflammation usually occurs to protect the liver from injury with characteristic of leukocytes infiltration such as neutrophils, monocytes, and lymphocytes. However, once this process is excessive, prolonged, or dysregulated, pathological inflammation and tissue injury will occur, which is critical for the initiation and development of hepatitis, fibrosis, cirrhosis, and liver cancer [30–32]. Hepatic inflammation has dual roles in the liver; they are essential to maintain tissue healthy and act as critical drivers of the liver pathology when persisted or out of control. The infiltration of leukocytes is a complicated process involving participation of many receptors, adhesion molecules, and chemokines, such as selectins, intercellular adhesion molecule-1 (ICAM-1), vascular cell adhesion molecule-1 (VCAM-1), and corresponding leukocyte receptors and monocyte chemoattractant protein 1 (MCP-1). In addition to hepatocyte death-mediated leukocytes infiltration, infiltrated inflammatory cells also produce soluble mediators, such as metabolites of arachidonic acid, cytokines, and chemokines, which activate related signal transduction cascades and change transcription factors, to further recruit inflammatory cells to the injured site. Then, liver injury is deteriorated because of increased cytokines and ligands [6, 33].

Similar to oxidative stress, inflammation is generally sustained and participated in the whole spectrum of liver diseases from initial to advanced stage, which is known as inflammation-fibrosis-cancer axis [8]. With the progression to chronic liver diseases, both innate and adaptive immune response are triggered by leukocytes infiltration, activation of Kupffer cells (KCs), and upregulation of inducible nitric oxide synthase (iNOS) [34]. Leukocytes and KCs produce huge amounts of nitric oxide and cytokines, such as potent profibrogenic cytokine, TGF-*β*4, and inflammation modulator, TNF-*α*. Plentiful inflammatory mediators including inflammatory cytokines, chemokines, and Toll-like receptors (TLRs) are involved in the regulation of hepatic fibrogenesis [34–36]. Particularly, TLRs, a family of pattern recognition receptors serving as important innate immune response factors, play a vital role in the pathogenesis of liver disease [37]. As Toll-like receptors are redox-sensitive receptor proteins and have been involved in cellular response to oxidative stress, the effect of lipid peroxidation caused by oxidants on TLRs is worth to be explored in the future. As a matter of fact, NF-*κ*B and JNKs are considered as the most key signaling pathways linking inflammation and fibrosis. Hyperactivation of NF-*κ*B in hepatocytes or infiltrated inflammatory cells fosters hepatic inflammation by increased generation of proinflammatory cytokines, such as IL-1*β*, TNF-*α*, and IL-6 [16]. Mediators like IL-1*β* and TNF-*α* could activate NF-*κ*B in hepatic stellate cells (HSCs) and promote survival of HSCs and fibrogenesis. Recently, it was discovered that ubiquitin-editing protein A20, an important regulator of inflammatory signaling to block NF-*κ*B activation, prevents the development of chronic hepatic inflammation and cancer by protecting hepatocytes from death [38]. JNK is involved in multiple signaling cascades related with hepatocellular injury, as well as regulating hepatic steatosis and inflammatory gene expression. It has direct profibrogenic role by stimulating platelet-derived growth factor (PDGF), TGF-*β*, and angiotensin II-induced proliferation, *α*-smooth muscle actin (*α*-SMA) expression, and collagen production [6, 39, 40]. Moreover, inflammation is intensively implicated in carcinogenesis of HCC [41]. Chronic inflammation is involved in the process of cellular transformation, promotion, proliferation, invasion, angiogenesis, and metastasis of carcinogenesis [42]. The generation of proinflammatory cytokines like cyclooxygenase-1 (COX-1), COX-2, TNF-*α*, IL-1, IL-26, IL-8, IL-18, and macrophage inflammation protein-1 (MIP-1*α*) via activation of NF-*κ*B alters the hepatic microenvironment and leads to fibrosis and carcinogenesis [24, 42].

## 3. The Relationship and Interdependence between Oxidative Stress and Inflammation in Various Liver Diseases

**Figure 1:**
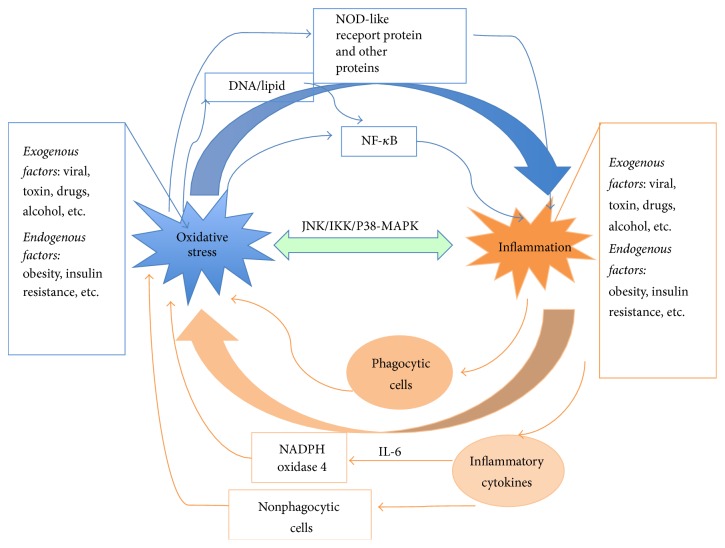
Potential interaction of oxidative stress and inflammation.

Extensive research has revealed that oxidative stress and inflammation are tightly interrelated in many diseases, as displayed in Figure 1. They seem to occur simultaneously and further promote each other in injury site. On one hand, continued oxidative stress can lead to chronic inflammation. Many transcription factors and receptors such as NF-*κ*B, activator protein-1 (AP-1), p53, hypoxia-inducible factor 1-alpha (HIF-1*α*), PPAR-*γ*, *β*-catenin/Wnt, and erythroid 2-related factor 2 (Nrf2) are activated by oxidative stress, which could regulate the expression of many genes, including those inflammatory cytokines and anti-inflammatory molecules [43]. For example, a pattern recognition receptor, NOD-like receptor protein 3 (NLRP3) that triggers innate immune response through promoting maturation of proinflammatory cytokines like IL-1*β* and IL-18, can be activated by oxidative stress and thus leads to inflammation [7, 43, 44]. Of note, NF-*κ*B signaling plays a key role in oxidative stress-mediated inflammation response [16]. Apart from the direct activation of NF-*κ*B via oxidative stress, damaged DNA induced by ROS also results in inflammation through NF-*κ*B pathway [16]. Recently, it was found that the release of oxidized peroxiredoxin-2 (PRDX2) and substrate thioredoxin from macrophages could alter the redox status of cell surface receptors and induce inflammation via TNF-*α* production in an oxidative cascade [45], which provides a new linkage of inflammation and oxidative stress. On the other hand, sustained inflammation could induce oxidative stress. Infiltrated immune cells and activated phagocytic cells such as neutrophils and macrophages produce large amounts of ROS and RNS like superoxide, hydrogen peroxide, hydroxyl free radical, peroxynitrite, and nitric oxide. These reactive species, which are generated to invade agents, can induce localized oxidative stress when diffused out of the phagocytic cells [44, 46]. In addition to direct production of ROS/RNS by phagocytic cells, in response to proinflammatory cytokines such as IL-6 and interferon-gamma (IFN-*γ*), the nonphagocytic cells can also produce reactive species. Redox-sensitive signal transduction pathways such as JNK and p38 MAPK also play vital roles in the interaction between inflammation and oxidative stress [14].

### 3.1. Alcoholic Liver Disease

As mentioned before, both generation of ROS/RNS and activation of inflammation are critical for hepatic damage induced by alcohol. The oxidative microenvironment created by ROS/RNS via alcohol metabolism process activates stress-related proteins, facilitates adduct formation, and induces endoplasmic reticulum stress, resulting in hepatocellular damage [47]. Particularly, the contributions of oxidative stress in promoting adaptive immune responses in alcoholic liver disease (ALD) have been reported [21, 48]. Oxidative stress has been considered as a trigger for adaptive immune responses in patients of alcoholic liver disease. The adaptive immune responses to MDA adducts indicates that oxidative stress may represent an important stimulus for the development of immune responses associated with advanced ALD [21]. The infiltration of inflammatory cells, activation of macrophage, and the proinflammatory mediators like LPS, TNF-*α*, IL1*β*, and IL6 induced by various pathways, such as alcohol-regulated CD14/Toll-like receptor 4 (TLR4) pathway, could provoke cellular injury and apoptosis, resulting in ALD [18, 19]. The activation of KCs by lipopolysaccharides (LPS) via TLR4 is critical to the onset of alcohol-induced hepatic damage, which could enhance the production of inflammatory cytokines and ROS which contribute to ALD [31, 49, 50]. MicroRNA-155, an important regulator of inflammation, has been demonstrated to play a vital role in alcohol-induced steatohepatitis and fibrosis. It was found that miR-155 knockout mice are protected from alcohol-induced steatosis and inflammation, which might be attributed to increased peroxisome proliferator-activated receptor response element (PPRE) and PPAR-*α* binding and decreased MCP1 production [51]. In the past, the role of oxidative stress and inflammation in the pathophysiological process of ALD was investigated individually. However, of note, with better understanding of this disease, recent studies have begun to explore the interconnected relationship between oxidative stress and inflammation [10].

**Figure 2:**
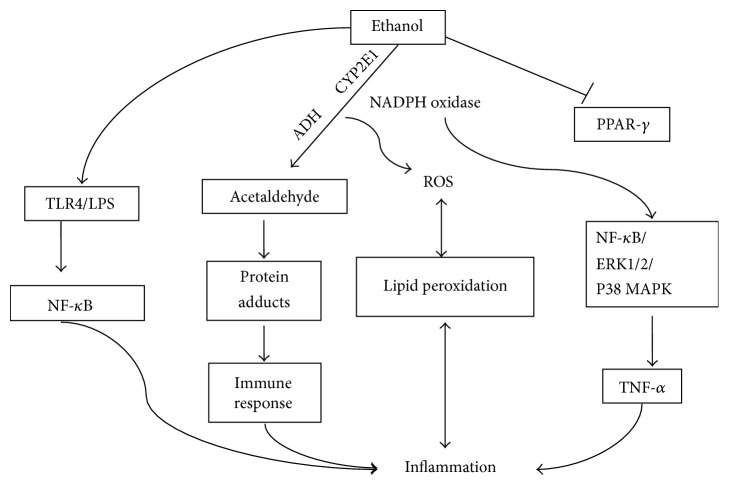
Several signaling pathways involved in oxidative stress-mediated aggravated inflammation in ALD.

Several signaling pathways have been identified to be involved in oxidative stress-mediated aggravated inflammation in ALD as shown in Figure 2. The I*κ*B kinase (IKK) kinase activation that leads to the production of proinflammatory cytokines could be accentuated by oxidative stress in ALD, for example, ROS-mediated molecular chaperones such as Hsp90 [31, 52–54]. Notch1 and NF-*κ*B that have been intensively implicated in inflammatory diseases were found to be important mediators in oxidative stress-induced alcoholic steatohepatitis [55]. NADPH oxidase, a source of ROS in ALD, can increase NF-*κ*B activation and phosphorylate ERK1/2 and p38 MAPK kinases that intensify the production of TNF-*α* from KCs [53]. In previous study, the role of alcohol-induced oxidative stress in modulating proinflammatory cytokines production in alcoholic steatohepatitis was determined. It was found that LPS improved TNF-*α*, macrophage inflammatory protein-alpha (MIP-1*α*), MCP-1, and cytokine-induced neutrophil chemoattractant 1 (CINC-1) in KCs-SV40, whereas TNF-*α* upregulated CINC-1, IFN-*γ*-inducible protein 10 (IP-10), and MIP-2 expression in H4IIEC3 hepatoma cells in a dose-dependent matter. When stimulated by combination of hydrogen peroxide with LPS or TNF-*α*, KCs-SV40 and hepatocytes increased production of proinflammatory cytokines through NF-*κ*B activation and histone H3 hyperacetylation. But regarding LPS-treated KCs-SV40, 4-hydroxynonenal showed inhibitory effect on cytokine production via significantly enhancing mRNA degradation of cytokines like TNF-*α*, MIP-1*α*, and MCP-1 and decreased the MCP-1 protein level by diminishing the phosphorylation of mRNA binding proteins [56]. This study indicates that the role of oxidative stress in regulating inflammatory cytokine production is dependent on cell type, while NF-*κ*B signaling, histone acetylation, and mRNA stability are implicated in this regulation process [56]. Interferon regulatory factor 3 (IRF3) and signal transducer and activator of transcription 3 (STAT3) have also been proposed to increase hepatic proinflammatory cytokines in ALD in association with oxidative stress, which needs further study to confirm. These signaling pathways are of great value to be specifically targeted for ALD treatment.

### 3.2. Nonalcoholic Fatty Liver Disease

Nonalcoholic fatty liver disease (NAFLD), which is characterized by excessive lipid accumulation in the liver, is similar to ALD that may progress from simple steatosis to steatohepatitis, fibrosis, cirrhosis, or even HCC [57, 58]. However, unlike ALD which is induced by heavy alcoholic consumption, the major etiology of NAFLD is obesity, insulin resistance (IR), dyslipidemia, or diabetes [52, 59]. Due to high caloric diet and life style, NAFLD has an increasing prevalence in Western society and even in the world. In the regard of pathogenesis of NAFLD, two hit hypotheses are widely proposed. It is believed that steatosis resulted from accumulated triglycerides and free fatty acid in the liver because of obesity or insulin resistance is the first hit, which might be a benign and stable pathology [60, 61]. There exist other pathological “hits” including oxidative stress and hepatic inflammation which may promote simple steatosis to further complications like fibrosis or cirrhosis [31]. Therefore, the importance of oxidative stress and inflammation in the pathogenesis of NAFLD is beyond doubt.

In nonalcoholic steatohepatitis (NASH), ROS are generated via a variety of ways [62]. *β*-Oxidation of overload fatty acid is regarded as the major source of reducing equivalents responsible for increased ROS production. Also, TNF-*α* and lipid peroxidation products, which inhibit the electron-transport chain of the mitochondria, could induce mitochondrial dysfunction and increase the production of ROS [63]. Mitochondrial damage will result in secondary inhibition of lipids *β*-oxidation and further increase the level of steatosis. In the cytosol, xanthine oxidase, a key enzyme for the degradation of purine, catalyzes the reaction of hypoxanthine to uric acid in which large amounts of superoxide anions are generated. Increased xanthine oxidase activity induced by 4-HNE may cause further hepatic damage via ROS [64]. The microsomal cytochromes CYP2E1 and CYP4A are involved in the lipooxygenation of long chain fatty acids with generation of ROS [65]. Moreover, CYP2E1 can be upregulated by free fatty acids as well as insulin resistance, which could enhance NADPH oxidase enzyme, leading to increased production of superoxide [66, 67]. In terms of inflammation, infiltration of various inflammatory cells like neutrophils, macrophages, T helper cells, natural killer T (NKT) cells, and natural killer (NK) cells and upregulation of inflammatory mediators such as TNF-*α*, IL-6, and IL-1*β* occur in steatohepatitis, which contributes to insulin resistance as well as other metabolic dysregulations, and play a key role in promoting the progression of steatohepatitis to fibrosis, cirrhosis, and cancer [32]. For example, lipid peroxidation-activated KCs could induce production of TNF-*α*. In the initial phase of NAFLD, resident immune cells such as KCs and dendritic cells respond to early signs of hepatocellular damage by generating plentiful proinflammatory cytokines like IL-1*β* and chemokines like chemokine ligand 2 (CCL2), which further promote inflammatory cell infiltration, thus leading to a vicious cycle [64, 68]. It was reported recently that early depletion of KCs using liposomal clodronate could block the development of NASH [69]. In addition, several mediators have been demonstrated which play a positive role in regulating inflammation in the context of NAFLD. For example, IL-15 upregulates expression of chemokines like CCL2, CCL5, and CXCL10 and increases infiltration of mononuclear cells, promoting inflammation in NAFLD [70]. Tumor necrosis factor receptor-associated factor 1 (TRAF1), an important adapter protein, is extensively implicated in mediating immunity/inflammation and cell death. It was reported recently that TRAF1 functions as a positive regulator of inflammation and hepatic steatosis in NAFLD through the activation of ASK1-P38/JNK axis [71]. Moreover, component C5, a central mediator of inflammation, was also found to contribute to liver steatosis and inflammation in NAFLD [72].

**Figure 3:**
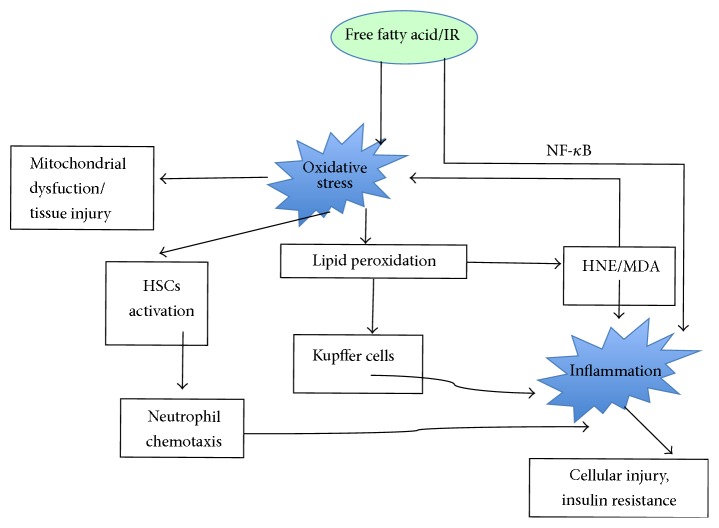
Potential interactions between oxidative stress and inflammation in the context of NAFLD.

Accumulating evidences showed tight interaction between oxidative stress and inflammation in the context of NAFLD (Figure 3). Firstly, ROS can induce lipid peroxidation process in which HNE and MDA are generated, which can freely diffuse into the extracellular space to affect nucleotide and protein synthesis and thereby increase proinflammatory cytokine and activate hepatic stellate cells, ultimately leading to inflammation and the progression of NASH [73, 74]. It was found that the levels of markers of lipid peroxidation and oxidative DNA damage, such as HNE and 8-hydroxydeoxyguanosine, are correlated with the severity of necroinflammation and fibrosis in patients with NAFLD [61, 75]. Lipid peroxidation products developed from phospholipids oxidation can induce adaptive immune responses by forming immunogenic adducts through the interaction with cellular proteins. High titres of antibodies against lipid peroxidation-derived antigens are associated with increased hepatic inflammation and advanced fibrosis in patients with NAFLD [76, 77]. Specifically, ROS and lipid peroxidation induce inflammation through promoting the release of proinflammatory cytokines, resulting in neutrophil chemotaxis and lesions of NASH [23]. For example, ROS activate NF-*κ*B signaling pathway, leading to the synthesis of TNF-*α* and the upregulated TGF-*β*, IL-8, IL-6, and Fas ligand. Recently, Satapati et al. reported that the induction of biosynthesis via hepatic anaplerotic/cataplerotic pathways is amplified by increased oxidative metabolism and further contributes to oxidative stress and inflammation during NAFLD [78]. Furthermore, TGF-*β*, IL-8, and HNE are chemoattractants of human neutrophils, which may result in more neutrophil infiltration. On the other hand, infiltrated neutrophils and other immune cells produce more ROS in hepatic lesion. Moreover, increased proinflammatory mediators such as TNF-*α* also cause the increase of ROS, leading to mitochondrial dysfunction [74, 79, 80].

### 3.3. Liver Fibrosis

Liver fibrosis, a reversible multicellular wound healing process that results from chronic liver injuries with excessive collagen and extracellular matrix (ECM), is characterized by perpetuation of parenchymal necrosis, infiltration of inflammatory cells, and activation of HSCs, macrophages, and KCs, regardless of the etiology [25, 81, 82]. Various growth factors, inflammatory cytokines and chemokines, accumulated ECM, and oxidative stress have been proposed to play a role in fibrogenesis.

Oxidative stress-related molecules and pathways can modulate tissue and cellular events involved in the pathogenesis of liver fibrosis [24]. The presence of oxidative stress and decreased antioxidant defenses caused by stimulus has been detected in almost all settings of fibrosis and cirrhosis in clinical and animal model. The disruption of lipids, proteins, and DNA caused by oxidative stress will induce necrosis and hepatocytes death and intensify the inflammatory response, resulting in the initiation of fibrosis [83]. Those ROS can stimulate the production of profibrogenic mediators from infiltrated inflammatory cells. Remarkably, ROS can interact directly with HSCs, which are the main executors of fibrogenesis to generate ECM. The cellular redox environment could regulate the entry of quiescent HSCs into activated cycle [24, 84]. Redox-sensitive transcription factors such as NF-*κ*B are important to regulate the activities of antioxidant enzymes that mediate ROS signaling. Compared with activated HSCs, the expression of NF-*κ*B in quiescent HSCs is lacking, implying that a redox-sensitive activation of NF-*κ*B can regulate expression of related genes, delivering a proper cellular redox microenvironment for quiescent HSCs into the proliferative cycle [24, 35, 85]. Phagocytosis of apoptotic bodies by HSCs induces NADPH with production of oxidative radicals, which is implicated in liver fibrosis in vivo [86]. Furthermore, ROS can also interact with HSCs to activate fibrosis by DNA methylation, histone modifications, and gene silencing by noncoding RNA species. The adaptive immune response induced by oxidative stress like lipid peroxidation products like MDA and 4-HNE was also implicated in the development from fatty liver to fibrosis. It was suggested that the adaptive immune reactions triggered by oxidative stress could be an independent predictor of progression of NAFLD to advanced fibrosis [76].

In the initial phase of hepatic injury, inflammation triggered by initial cell death contributes to the removal of cellular debris and promotes liver regeneration, ensuring restoration of hepatic architecture and function after acute liver injury [87, 88]. However, once underlying disease cannot be eliminated and the stimuli sustains, chronic inflammation and progressive liver fibrosis will be induced [40]. As a matter of fact, cell death and persisted overwhelming inflammation are the characteristics of chronic liver diseases that progress to fibrosis. A variety of inflammatory mediators and pathways could regulate the activation of HSCs and their survival after activation [89]. It has been found that inflammatory cytokines like IL-1*β*, TNF-*α*, and IL-17/20/33, chemokines like MCP-1 and CXCL10, and TLR pathway are intensively involved in the regulation of hepatic fibrogenesis [90–93]. T cells response also plays a vital role in regulating the progression of fatty liver to advanced-stage liver disease [94–96]. It has been indicated that hepatic recruitment of T helper cells and cytotoxic T cells contributes to hepatic inflammation, leading to the development from simple fatty liver to steatohepatitis [97]. T helper cells and cytotoxic T cells infiltration was correlated with the progression of NASH, paralleling the worsening of parenchymal injury and lobular inflammation [96]. CD4+ T helper cells may promote hepatic inflammation through upregulation of IFN-*γ* and CD40 ligand [98]. But the underlying mechanism of CD8+ cytotoxic T cells in promoting NASH progression is still unclear.

**Figure 4:**
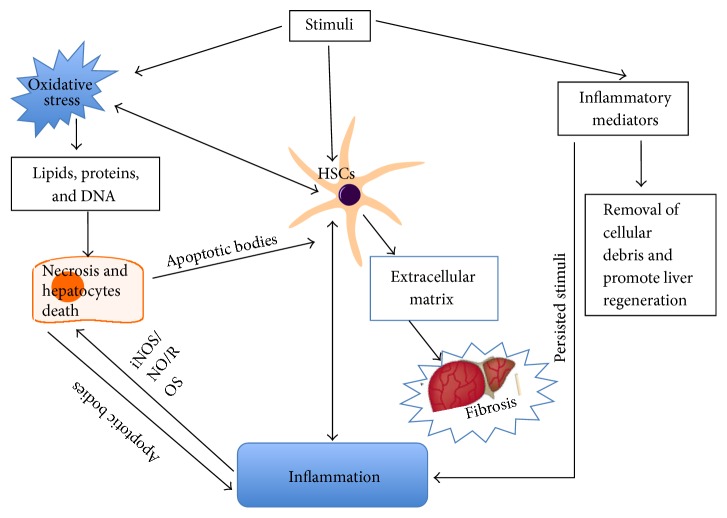
Oxidative stress and inflammation interact with each other in multiple ways to promote fibrogenesis.

Thus, oxidative stress and inflammation interact with each other in multiple ways, thereby creating a favorable microenvironment for fibrogenesis (Figure 4). In addition to direct interplay of ROS and inflammation, the activated HSCs are a vital bridge to link them together. Both oxidative stress and inflammatory mediators play a role in the activation of HSCs, and, conversely, activated HSCs could in turn enhance cellular oxidative stress and inflammation. On one hand, redox-sensitive pathways influenced by oxidative stress regulate the status of HSCs in the initiation phase and stimulate inflammatory signaling via cytokines, chemokines, and TLR ligands [25, 99]. On the other hand, HSCs activated by inflammatory mediators could also inhibit antioxidant defense and increase the generation of ROS. They work tightly to form a vicious cycle in the process of liver fibrosis. Although there are no highly effective antifibrogenic agents currently available, the potential candidates that can reduce inflammation and oxidative stress as well as ECM are considered to be promising for the prevention and treatment of liver fibrosis. Combination therapy that blocks HSCs activation via antioxidant and anti-inflammatory pathways would be effective to inhibit the progression of the pathogenesis of liver fibrosis.

### 3.4. Liver Cancer

The initiation and progression of many cancers have been linked to oxidative stress and inflammation, as demonstrated by the excessive sustained ROS and inflammatory mediators. Primary liver cancer, the fifth most common malignancy worldwide, has no exception. Sustained inflammation is associated with persistent hepatic injury and concurrent regeneration, leading to sequential development of advanced stage of liver disease including fibrosis, cirrhosis, and eventually HCC [100]. The perpetuation of a wound-healing response activated by hepatocytes death and the following inflammatory cascade response is a common denominator for HCC initiation. Chronic inflammation has been linked to multiple steps involved in carcinogenesis, such as cellular transformation, promotion, invasion, angiogenesis, and metastasis [100, 101]. For example, in context of hepatic steatosis induced by obesity or other factors, excessive free fatty acids increase proinflammatory cytokines and adipokines that can further promote release of TNF-*α* and IL-6 from KCs, leading to activation of downstream signaling molecules like STAT3 in hepatocytes, which might result in hepatocarcinogenesis [102–105]. Meanwhile, ROS are produced over a long time under a sustained environmental stress and contribute to cellular structure damage and induce DNA damage and mutations in protooncogenes and tumor-suppressor genes, leading to neoplastic transformation. Under hypoxic conditions, excessive RNS are generated through the mitochondrial respiratory chain. The role of oxidative stress in modulating inflammation-induced carcinogenesis in different stage has been systemically reviewed by Reuter et al. Briefly, under an inflammatory stimulus, progression of carcinogenesis mediated by ROS may be direct through oxidation or nitration of DNA or mediated by the cross-talk signaling pathways related to oxidative stress and inflammation. The sustained inflammatory/oxidative microenvironment forms a vicious circle, which can damage healthy cells like neighboring epithelial and stromal cells, ultimately resulting in carcinogenesis.

Of note, the participation of ROS in carcinogenesis is complicated. ROS are regarded as tumorigenic due to their ability to increase cell proliferation, survival, and cellular migration as well as gene mutations. However, ROS can also function as antitumorigenic agents via induction of cellular senescence and cell death. Whether ROS promote tumor cell survival or induce cancer cell death remains as a complex issue, which majorly depends on the location of ROS production, the concentration of ROS, and many other factors such as carcinogenesis stage [15, 29]. For example, cytoglobin (*Cygb*), expressed in HSCs, plays a protective role in controlling ROS/RNS in the inflamed liver. Deficiency of* Cygb* promoted HCC development in CDAA-fed *Cygb* ^−/−^ mouse via upregulating prooxidative genes and downregulating antioxidative genes [28]. However, ROS produced by NOX2 could inhibit proliferation of cancer stem cell-like phenotype and diminish tumor growth of HCC by PPAR-*γ* agonists, suggesting their protective role in the context of cancer.

## 4. Immune Suppression: A Potential Linkage of Oxidative Stress and Inflammation

### 4.1. Immune Suppressive Cells to Resolve Inflammation

As aforementioned, hepatic inflammation has dual roles; they are essential to maintain tissue healthy and act as critical drivers of the liver pathology when persisted or out of control. Therefore, resolving inflammation appropriately and timely is of great significance to maintain liver homeostasis. In this regard, several basic mechanisms in resolution of inflammation were proposed. First, soluble anti-inflammatory mediators are released in order to fight against the innate immune response [39]. The second mechanism mainly involves the activation and induction of immune suppressive cells like regulatory T cells (Tregs). Additionally, the loss of cell-cell mediated costimulation on lymphocytes or antigen presenting cells (APC) and the activation of programmed cell death in those cells are also regarded as potential mechanisms to resolve inflammation. In particular, recently, the activation and induction of immune suppressive cells in resolving inflammation have been intensively studied and attracted great attention [107]. Herein, immune suppressive cells including Tregs, M2-macrophage, and myeloid-derived suppressor cells (MDSCs) are particularly discussed with emphasis on anti-inflammatory effects and linkage to oxidative stress on liver diseases.

#### 4.1.1. Regulatory T Cells

Tregs, a subset of T lymphocytes that primarily works at sites of inflammation in maintaining peripheral tolerance, have been implicated in several inflammatory liver diseases. Tregs are equipped with a wide range of mechanisms of immune suppression, including the removal of target cells, the regulation of APC, the disturbance of metabolic pathways, and the generation of anti-inflammatory cytokines [108]. Increasing evidences showed that the reduced frequency and defective function of Tregs facilitate inflammation in various liver diseases, such as drug induced liver injury, autoimmune hepatitis, and NASH.

Accumulating evidence indicates that Tregs are vital inhibitory mediators of inflammation. It has been demonstrated that, in the initial phase of acute liver injury, intrahepatic Tregs diminished promptly through apoptosis, which may facilitate inflammation and tissue injury, while in the healing phase, Tregs are generated through matrix metalloproteinase (MMP) cascade-dependent activation of TGF-*β* to terminate inflammation [109]. Development from NAFLD to NASH is marked by an increased frequency of Th17 cells in the liver, which is a subset of proinflammatory T helper cells with the production of IL-17 and is tightly related to Tregs because the signals for Th17 cells differentiation could inhibit Tregs. The ratios of Th17/resting Tregs in peripheral blood and in the liver were also increased [96]. Furthermore, they inhibit the profibrogenic inflammatory milieu through suppressing the infiltration of profibrogenic CD8+ and IL-17+ T cells [110]. In regard of migration of Tregs, it was found that local proinflammatory cytokines lead to the secretion of CXCL9 and CXCL10 by sinusoidal and parenchyma cells, which could recruit CXCR3^high^ circulating Tregs into the liver. Subsequently, CCR4 guides the migration of these Tregs within the inflamed liver. These infiltrated Tregs respond to CCL17 and CCL22 released by activated DCs, thus resulting in their accumulation in the liver with dendritic cells-rich inflammatory infiltrates. The reason why inflammation persists in the presence of Tregs infiltration might be due to their dysfunctional suppressive effects by programmed death receptor-1 in the specific microenvironment [111].

Therefore, modulation of Tregs by immune regulatory agents or adoptive transfer is of great importance in inflamed liver diseases. For instance, dietary n-3 polyunsaturated fatty acids (PUFA) protected mice from Con A-induced hepatitis by enhancing Tregs generation via upregulated expression of PPAR-*γ* and TGF-*β*, which might be a promising potential therapeutic agent having anti-inflammatory and immunoregulatory effects for inflammatory diseases [112]. The population of Tregs in the liver significantly decreased after reperfusion, and adoptive transfer of induced Tregs (iTregs) that come from TGF-*β*-induced CD4+CD62L+T cells before ischemia reperfusion could attenuate liver injury as indicated by reduced proinflammatory cytokines. In vitro study showed that iTregs could suppress expression of IL-1*β* and TNF-*α*, promote transcription of IL-10, and increase phosphorylation of mothers against decapentaplegic homolog 3 (SMAD3) in KCs. Furthermore, inhibition of TGF-*β* signaling by anti-TGF-*β* abolished the effects on KCs [113]. In acetaminophen- (APAP-) induced liver injury, the depletion of Tregs amplified proinflammatory cytokines and aggravated liver injury, while adoptive transfer of Treg cells showed protective effect [114].

However, Tregs have also been proposed as foe in several types of liver diseases such as vital hepatitis and HCC, in which persisted immune responses are expected to eliminate exogenous infection and neoplasm [115–118]. Failure in immune regulation by Tregs leads to viral persistence and tumor growth. Therefore, appropriate strategy in regulating population and function of Tregs particularly in liver diseases should be highlighted according to different conditions.

#### 4.1.2. Alternatively Activated (M2) Macrophage

There are two major phenotypes of macrophages: classically activated (M1) subset with prototypic macrophage functions which acts as proinflammatory mediator and an alternatively activated (M2) subset involved in wound healing with anti-inflammatory ability. Therefore, macrophage polarization plays a critical role in inflamed liver diseases [119]. In patients with minimal liver damage and steatosis, there is higher hepatic expression of M2 genes compared to patients with more severe liver lesions. A complicated interplay between M1 and M2 types of macrophages expressing a wide range of molecules and receptors is involved in many liver diseases [120].

In ALD, both activated M1 and M2 macrophages are present in the liver [121]. The polarization of KCs toward M1 phenotype initiated the inflammatory process, and it was indicated that promoting anti-inflammatory M2 phenotype polarization protects liver from alcoholic-induced liver injury through mechanism that relies on apoptotic effects of M2 subset towards their M1 counterparts [121]. In addition, M2 macrophages could trigger hepatocyte senescence mediated by IL-6 and enhance alcohol-induced hepatocyte senescence by oxidative stress, which exhibit functional resistance to apoptosis, thus leading to an early protective effect against ALD [122]. In mice with chronic alcohol treatment, genes related with M1 phenotype such as TNF-*α*, MCP1, and IL-1*β* and genes associated with M2 macrophages such as Arg1, Mrc1, and IL-10 as well as the population of CD206(+)CD163(+) M2 macrophages were improved in the liver. Alcohol could promote M2 phenotype and the expression of Krüppel-like factor 4 (KLF4), a regulator of macrophage polarization, whereas the intermediate metabolite of alcohol, acetaldehyde, decreased KLF4 and promoted M1 macrophage, which may justify the increased M1 and M2 macrophages in ALD [123]. M2 macrophages expressing CD163 in liver sinusoids of ALD are abundant; IRF-4, which is related to IL-4 production, and M2 polarization were also observed in AH, suggesting that M2 phenotype plays a role in AH pathogenesis [120]. In TAA-induced liver injury model, the double labeling of CD68(+) and CD163(+) macrophages was found, which indicated that macrophage immunophenotypes are interchangeable in injured sites [124].

Pharmacologic interventions targeting M2 polarization during the early stages of liver disease may signify a striking strategy to alleviate liver injury [125, 126]. IL-6, a pleiotropic interleukin that commonly associated with proinflammatory effect, is also necessary for inflammation resolution because of its role in promoting polarization of M2 macrophage [119]. Despite the fact that it is associated with the perpetuation and development of inflammatory disease, the potential benefit of IL-6 in polarization of M2 macrophage should also be considered [119].

#### 4.1.3. Myeloid Derived Suppressor Cells (MDSCs)

The myeloid-derived suppressor cells (MDSCs) are a heterogeneous population from bone marrow with remarkable immunosuppressive properties. In the context of tumor setting, MDSCs help tumor cells escape from immunosurveillance, thereby endorsing tumor growth [127]. Both innate immune and adaptive immune responses could be suppressed by MDSC via several mechanisms including production of large amounts of nitric oxide (NO) as well as ROS and arginine depletion. However, their functional significance in the immune system has been appreciated in recent years [128–131].

Under most of the inflammation conditions, MDSCs infiltrate into the liver and play a protective role in hepatitis models. In models of acute hepatitis mediated by Con A and *α*-GalCer, farnesoid X receptor (FXR) activation drives the accumulation of MDSCs to liver via upregulation of S100A8 and augments the suppressive function of MDSCs through upregulation of receptor paired immunoglobulin-like receptor B (PIR-B) by binding the PIR-B promoter [132]. MDSCs function as an important negative feedback loop and reduction in this cell population facilitates inflammatory hepatic damage [133]. In the context of immune-mediated hepatic injury, mTOR negatively regulates the recruitment of MDSC, which is critically required for protection against hepatic injury. The inhibition of mTOR by rapamycin or other inhibitor treatments promotes the expansion of MDSCs that protect liver from persistent inflammation [134, 135].

Intervention targeting the expansion and activation of MDSCs is a potential therapeutic approach because deficient or unsuitable activity of this cell population may contribute to the pathogenesis of inflammatory liver. It was proposed that expansion of MDSC could be divided into two processes regulated by different signal transduction pathways. The first process is the expansion induced by various cytokines and growth factors produced in response to chronic stimulation, which involves factors like IL-6 and VEGF, and signaling pathways such as STAT3 and NF-*κ*B, which prevents maturation of myeloid cells. Then, a second activating signal provided by proinflammatory molecules to drive upregulation of arginase and iNOS and production of immune suppressive cytokines is required for their activation [130, 131]. In addition to above proposed pathways, it is reported that activated human HSCs during chronic inflammation induced mature peripheral blood monocytes into MDSCs, and, subsequently, excessive liver injury might be prevented by local induction of MDSCs. However, of note, in liver cancer patients, the expansion of HSC-induced MDSCs plays an opposite role to facilitate tumor growth by immune suppression [130, 131, 136].

### 4.2. Oxidative Stress Mediating Failure of Immune Suppression Response to Promote Inflammation

After unraveling the role of immune suppression in mediating inflammatory liver diseases, the question that whether oxidative stress, the essential partner of inflammation, participates in this process has been put forward. As oxidative stress and inflammation tightly interplay and drive coherently the progression of liver disease together, it is reasonable to propose that oxidative stress plays a critical role to promote inflammation in terms of negatively regulating the immune suppression. That is, downregulated immune suppression might be another potential linkage between oxidative stress and inflammation. As a matter of fact, increasing evidence demonstrated that oxidative stress could mediate the dysfunction of immune suppression, thus leading to the failure of inflammation resolving.

In a study of high-fat fed mice model, it was indicated that the increased oxidative stress in a fatty liver caused the apoptosis of Tregs and reduces the population of hepatic Tregs, resulting in lowered suppression of inflammatory responses. The treatment with an antioxidant Mn(III)tetrakis(4-benzoic acid)porphyrin chloride decreased the apoptosis of Tregs, increased the number of hepatic Tregs, and thus improved hepatic inflammation in mice with NAFLD. This scenario might be one of the underlying mechanisms that enable the conversion of simple steatosis into steatohepatitis [137]. In another study, it was indicated that monocytic MDSCs are induced by catalase-mediated exhaustion of hydrogen peroxide from mature CD14+ monocytes, and the frequency of MDSC inversely correlated with hydrogen peroxide level. The oxidative stress in many liver diseases with decreased activity of catalase and increased hydrogen peroxide might inhibit the expansion and activation of hepatic MDSCs that are expected to resolve overimmune response [138]. Therefore, oxidative stress might serve as a mediator of impeding immune suppression response to promote inflammation. This mechanism also probably explains the potential benefit of oxidative stress in tumor microenvironment. Therapy of antioxidant that activates immune suppression to resolve inflammation might be promising for treatment of various inflamed liver diseases.

## 5. Potential Therapeutic Approach on Liver Diseases

Resolving the vicious cycle between inflammation and oxidative stress is of great clinical importance to treat many chronic diseases. It was proposed that identification of primary abnormality is important to break this cycle. That is, in the case where oxidative stress presents as primary event, antioxidants rather than anti-inflammatory agents would be effective therapeutic strategy [7]. Conversely, if inflammation appears as the primary abnormality, anti-inflammation should be considered as a primary therapeutic target. It seems to be a key factor to explain the puzzling phenomenon of medical science, where, in certain case of oxidative stress-related disease, antioxidant treatment showed unsatisfactory outcome [7, 12]. However, despite promising potential for clinic practice, unraveling the primary abnormality is difficult as once the process has been initiated, both inflammation and oxidative stress show up to promote each other and to cause progressive injury. With regard to liver diseases, few researches were dedicated to study the causation of oxidative stress and inflammation in specific disease settings. Therefore, application of agents that modulate both oxidative stress and inflammation is still regarded as the mainstream choice for the prevention and treatment of liver diseases.

Many medicinal herbs show striking abilities in protecting the liver due to their remarkable anti-inflammatory and antioxidative effects. In recent years, traditional Chinese medicine (TCM) has received broad attention from the public due to its long-lasting curative effects and mild complications in treating a variety of liver diseases [178]. The applications of TCM in liver diseases have been systematically reviewed in our previous papers [2, 8]. In Table 1, agents including herbal medicine and derived compounds targeting oxidative stress and inflammation in various liver diseases were summarized. Particular emphasis herein is put upon several pure compounds from herbal plants with strong anti-inflammatory and antioxidative ability. Furthermore, several medicines that prevent liver disease by mechanism of regulating immune suppression response, antioxidant, and anti-inflammation were reviewed.

Berberine (BBR), an alkaloid isolated from* Coptidis Rhizoma*, has been reported with several pharmacological activities including antitumoral, antimicrobial, glucose- and cholesterol-lowering, and immunomodulatory properties by us and others [179–181]. Importantly, it exhibited antisteatotic, anti-inflammatory, and antioxidative effect via regulating AMPK and low-density lipoprotein receptor expression by ERK and JNK pathways [8]. BBR treatment significantly attenuated hepatic inflammation, lipid peroxides, and fibrosis in NAFLD model, which may be a therapeutic strategy to prevent the progress of hepatic steatosis to NASH [182]. In another study, it was shown that daily administration of BBR at the dose of 50 mg/kg for three weeks relieved oxidative stress, inflammation, hyperglycemia, hyperlipidemia, hyperinsulinemia, and the neurotoxicity related with NASH [183]. Pseudoberberine, a berberine analogue, displayed antioxidant and anti-inflammatory activities in diabetic mice with fatty liver. Due to poor bioavailability of BBR via oral administration, pseudoberberine might be a new oral hypoglycemic agent for NAFLD [184]. It has been reported that BBR ameliorated the liver fibrosis in mice with CCl4 administration in a dose- and time-dependent manner. The underlying mechanism of its protective effect on chronic liver fibrosis might be due to reduced oxidative stress, inhibition of TNF-*α*, COX-2, and iNOS expression, and activation of AMPK pathway [185, 186]. The effect of BBR on the drug isoniazid-induced hepatotoxicity has been conducted. The results showed that BBR protected liver from injury through upregulation of PPAR-*γ* and subsequently suppression of NF-*κ*B and iNOS and release of the proinflammatory cytokines [187]. In addition to isoniazid, BBR also alleviates cyclophosphamide-induced hepatotoxicity by modulating antioxidant defense and inflammatory mediators [188].

Curcumin, a polyphenolic antioxidative constituent in many plants, turmeric in particular, showed remarkable hepatoprotective effects. In a rat model with CCl4-caused liver injury, curcumin could effectively protect liver from fibrosis. The results showed that curcumin attenuates oxidative stress by upregulating hepatic glutathione level, leading to the reduction of lipid hydroperoxide. Additionally, curcumin intensely suppresses inflammation by diminishing contents of inflammatory cytokines, including IFN-*γ*, TNF-*α*, and IL-6. Furthermore, curcumin inhibits HSC activation by increasing the level of PPAR-*γ* and reducing platelet-derived growth factor and TGF-*β* as well as their receptors and type I collagen [189]. In rat model with thioacetamide- (TAA-) induced chronic hepatitis, hepatic levels of MDA, collagen deposition, inflammatory mediators, and liver function were improved by curcumin treatment [154]. The effect of curcumin on a drug gentamicin-induced liver injury was evaluated in a study. The results showed that liver function indicated as aspartate aminotransferase (AST), alanine transaminase (ALT), and lactate dehydrogenase (LDH) activities, liver histological alterations, antioxidant defense, inflammatory mediators like TNF-*α* level, proapoptotic proteins caspase 3 and Bax expression were significantly improved by curcumin [171]. Curcumin also displayed protective effects on tetrachloro-p-benzoquinone- (TCBQ-) induced hepatotoxicity in mice, which was demonstrated by the improved AST and ALT activities, and histopathological changes including centrilobular necrosis and inflammatory cells infiltration. The elevated TBAR level and the inhibited activities of SOD and catalase and upregulated iNOS, COX-2, IL-1*β*, IL-6, TNF-*α*, and NF-*κ*B induced by TCBQ were effectively reduced by curcumin through the activation of Nrf2 signaling [190].

Epigallocatechin gallate (EGCG) is a type of catechin in many natural plants such as green tea. As a polyphenol, it has a wide range of health benefits. For instance, EGCG could significantly relieve bile duct ligation-induced hepatic damage in mice via improving mitochondrial oxidative stress and inflammation. EGCG reduced gene expression of profibrotic markers like collagen, fibronectin, *α*-SMA, and connective tissue growth factor (CTGF), cell death marker like DNA fragmentation and PARP activity, mitochondrial oxidative stress, NF-*κ*B activity, and proinflammatory cytokines such as TNF-*α*, MIP1-*α*, IL-1*β*, and MIP2 in bile duct ligation mice. Mitochondrial electron transport chain complexes and antioxidant defense enzymes like GSH-Px and SOD were improved by EGCG administration [191]. In NAFLD model, treatment with EGCG improved fatty score, necrosis, and inflammatory foci, restored liver function, and reduced fibrogenesis with downregulation of nitrotyrosine formation and proinflammatory markers such as iNOS, COX-2, and TNF-*α*. The activity of TGF/SMAD, PI3K/Akt/FOXO1, and NF-*κ*B pathways could be counteracted by EGCG, suggesting that EGCG is beneficial in the prevention of NAFLD [149]. In addition, EGCG effectively weakened the severity of CCl_4_-induced liver injury and the progression of liver fibrosis by mechanism of the reduction in oxidative stress and the inflammatory response [192]. Furthermore, it was demonstrated that, even in healthy rats, EGCG extends lifespan by reducing liver function damage and attenuating age-associated inflammation and oxidative stress. EGCG inhibited NF-*κ*B signaling and increased the expressions of longevity factors including silent mating type information regulation 2 homolog 1 (SIRT1) and forkhead box class O 3a (FOXO3a) [193].

Lipoic acid (LA) is an organosulfur compound derived from octanoic acid, which has two sulfur atoms connected by a disulfide bond and thus can be oxidized, and the molecule presents as two enantiomers (S)-(−)-lipoic acid (SLA) and (R)-(+)-lipoic acid (RLA) and as a racemic mixture (R/S)-lipoic acid (R/S-LA). Its effect on many liver diseases has been widely studied. LA protects liver from aflatoxin B-1 induced injury via downregulating mRNA level of IL-6 and decreasing the protein expressions of both NF-kB p65 and iNOS in broilers [166]. In liver injury induced by LPS, a key inflammatory component of Gram-negative bacteria which contributes to the development of hepatic failure, administration of LA following LPS treatment effectively prevented oxidative stress and hepatic inflammation [194]. In another study, the protective role of LA in LPS/D-galactosamine- (D-GalN-) induced fulminant hepatic failure in mice was studied. It was found that ROS and TBARS were eliminated, while activity of hepatic CAT and GPx was increased by LA treatment. Additionally, pretreatment with LA significantly decreased LPS/D-GalN-induced expression of inflammatory mediators such as TNF-*α*, NF-*κ*B, iNOS, COX-2, IL-6, and IL-1*β* [157]. In rat model with TAA-induced chronic hepatitis, hepatic levels of MDA and liver function were improved by RLA treatment. The depletion of GSH, macrophage activation, collagen deposition, and expression of NF-*κ*B, TNF-*α*, and IL-6 were significantly decreased in response to RLA administration [154]. In addition, it was found that R/S-LA coadministration could effectively disrupt a vicious pathogenic circle constituting oxidative stress, insulin resistance, and inflammation induced by fructose in rats model [195].

## 6. Conclusion

The critical roles of oxidative stress and inflammation involved in the pathogenesis of liver diseases have been highlighted for decades, and accumulating evidence showed that a vicious cycle could be created by oxidative stress and inflammation, which participates tightly in the progression of liver diseases. Oxidative stress could elevate proinflammatory gene expression by signaling pathways such as NF-*κ*B, while infiltrated inflammatory cells and cytokines like IL-6 and IFN-*γ* could produce more oxidative stress. Their tight interactions make the hepatic pathological process complicated. More key interplayed molecules and targets are expected to be discovered in future studies. Importantly, as immune suppressive cells including Tregs, M2 macrophage, and MDSCs are of great importance to resolve inflammation, the failure of immune suppression leads to the sustained inflammation. Increasing evidence showed that immune suppression might be a link between oxidative stress and inflammation as indicated by the increased oxidative stress mediating inhibition of immune suppression in inflamed liver. Intervention mediating immune suppression might be promising to alleviate both inflammation and oxidative stress. Application of agents modulating both oxidative stress and inflammation is still considered to be mainstream choice for the prevention and treatment of liver diseases. Many medicinal herbs or derived compounds show striking abilities in protecting the liver due to their remarkable anti-inflammatory and antioxidative effects in animal studies. In future study, the related translational research should also be further conducted and improved to realize the application of these agents in treating and prevention of liver diseases. Factors such as the duration of treatment, dosage, bioavailability in human body, mode of administration, and rigorous clinical study design are needed to be considered. In short, intensive efforts should be made to address the vicious pathological cycle forming by oxidative stress and inflammation through interacted pathways or immune suppression.

## Figures and Tables

**Table 1:** Herbal medicines or derived compounds targeting oxidative stress and inflammation in various liver diseases.

Materials	Models	Effects	Mechanisms	Refs.
Phyllanthin	CCl4-induced hepatic toxicity	↓ oxidative stress and hepatic fibrosis	↓ TNF-*α*/NF-*κ*B and profibrotic factor TGF-1 mediating inflammatory signaling	[139]
Gomisin A	CCl4-induced acute liver injury in rats	↓ oxidative stress and inflammatory response; ↓ fibrogenesis	↓ NF-*κ*B and proinflammatory mediators	[140]
*Dillenia suffruticosa* leaves	CCl4-induced hepatic damage in rats	Impedes hepatic damage	↓ oxidative stress and inflammatory markers	[141]
Diallyl disulfide	CCl4-induced liver damage in rats	↑ phase II/antioxidant enzymes; ↓ inflammatory mediators	↑ Nrf2 pathway; ↓ NF-*κ*B activation	[142]
Folic acid and melatonin	CCl4-induced liver injury in rats	↑ liver function; ↓oxidative stress and inflammation in rats	Restore the oxidative stability and lipid profile; ↓ inflammatory cytokines and cell survival Akt1 signals	[143]
Ursolic acid	CCl4-induced liver injury in mouse	Inhibiting oxidative stress and inflammation	↓ JNK, p38 MAPK, ERK, and NF-*κ*B	[144]
Aloin	Chronic alcoholic liver injury in mice	↓ chronic alcoholic liver injury	↓ lipid accumulation, oxidative stress, and inflammation	[145]
Wild bitter gourd	Chronic alcohol-induced liver injury in mice	↓ oxidative stress and inflammatory responses	↑ antioxidant defence system; ↓ MDA and proinflammatory cytokines; ↓ CYP2E1, SREBP-1, FAS, and ACC protein expression	[146]
Lutein	Alcohol-induced liver injury in rats	↓ oxidative stress and inflammation	↓ inflammatory proteins and cytokines; ↑ Nrf2 levels; ↑ antioxidant enzymes	[147]
Fucoidan	High-fat diet-induced NAFLD in rats	↓ steatohepatitis and insulin resistance	↓ oxidative stress and inflammatory cytokines	[148]
Epigallocatechin gallate	High-fat diet-induced nonalcoholic fatty liver disease in rats	↓ fibrosis, oxidative stress, and inflammation	Modulating the activities of TGF/SMAD, PI3 K/Akt/FOXO1, and NF-*κ*B	[149]
Magnolia extract	High-fat diet-induced liver damage in mice	↓ fibrosis in the liver	Inhibition of lipid accumulation, inflammation, and oxidative stress	[150]
Brown alga *Ecklonia cava *polyphenol extract	High-fat diet-induced obese mice	↓ hepatic lipogenesis, oxidative stress, and inflammation	↑ AMPK and SIRT1	[151]
Chicoric acid	High-fat diet-fed mice	↓ hepatic steatosis, inflammation, and oxidative stress	↑ antioxidant defense system; ↑ adipocytokines and adipogenesis-associated proteins	[152]
Ghrelin	High-fat diet-induced NAFLD in rats	↓ inflammation, oxidative stress, and apoptosis	Restoration of LKB1/AMPK and PI3 K/Akt pathways	[153]
Curcumin, silybin phytosome, and R-lipoic acid	TAA-induced chronic hepatitis in rat model	↓ MDA, GSH depletion, and collagen deposition	↓ macrophage activation, NF-*κ*B, TNF-*α*, and IL6	[154]
Carvacrol	TAA-induced liver injury in rats	Abrogation of oxidative stress, inflammation, and apoptosis	↓ NF-*κ*B	[155]
Fermented rooibos extract	LPS-induced liver injury in rats	Attenuated liver injury	↓ oxidative stress and proinflammatory cytokines	[156]
*α*-Lipoic acid	LPS-induced liver injury	Antioxidant, anti-inflammatory, and antiapoptotic activities	↓ iNOS, COX-2, TNF-*α*, NF-*κ*B, IL-1*β*, and IL-6	[157]
Paeoniflorin	A mouse model of hepatic ischemia/reperfusion injury	↓ hepatic ischemia/reperfusion injury	Via antioxidative, anti-inflammatory, and antiapoptotic pathways	[158]
Agaricoglycerides	Hepatic ischemia/reperfusion injury in mouse	Protect against hepatic injury	↓ inflammatory response, oxidative stress, and expression of NF-*κ*B	[159]
Cannabidiol	Hepatic ischemia/reperfusion injury in mouse	↓ oxidative stress and inflammation and cell death	↓ key inflammatory pathways, independent of classical CB1/2 receptors	[160]
Dioscin	Hepatic ischemia/reperfusion injury in rats	Inhibition of oxidative-nitrative stress, inflammation, and apoptosis	↓ IL-1A, IL-6, TNF-*α*, intercellular adhesion molecule-1, MIP-1*α*, MIP-2, Fas, and FasL; ↓ NF-*κ*B, AP-1, COX-2; ↓ JNK, ERK, and p38 MAPKs phosphorylation; ↑ Bcl-2 and Bcl-x	[161]
Delta(8)-tetrahydrocannabivarin	Hepatic ischemia/reperfusion injury in mice	Prevents hepatic injury and inflammation	Cannabinoid CB2 receptors	[162]
Green tea extract	Hepatic injury after hemorrhage/resuscitation in rats	↓ apoptosis, oxidative stress, and inflammation	↓ JNK and NF-*κ*B	[163]
Astaxanthin and *Corni fructus*	Streptozotocin-induced diabetic rats	↓ glucose concentration; ↓ ROS and lipid peroxidation	↓ advanced glycation end product formation and anti-inflammation	[164]
Resveratrol	Streptozotocin-induced type 1 diabetic rats	↓ oxidative stress and inflammation	↑ Mn-SOD; ↓ NF-*κ*B and IL-1*β*;	[165]
*α*-Lipoic acid	Aflatoxin B-1-induced liver damage in broilers	↓ oxidative damage and inflammatory responses	↓ hepatic proinflammatory cytokines; ↓ NF-*κ*B	[166]
Niacin	HepG2 or human primary hepatocytes stimulated with palmitic acid	↓ fat accumulation, oxidative stress, and inflammatory cytokine IL-8	↓ hepatocyte DGAT2 and NADPH oxidase activity	[167]
Quercetin	Tripterygium glycosides-induced acute liver injury in mice	↓ liver injury	↓ oxidative stress and inflammation	[168]
Troxerutin	2,2′,4,4′-Tetrabromodiphenyl ether-induced liver inflammation in mouse	↓ liver inflammation	↓ oxidative stress-mediated NAD^+^-depletion	[169]
Geraniol	2-Acetylaminofluorene-induced liver injury in rats	↓ oxidative stress, inflammation, and apoptosis	↓ caspase-3 and caspase-9, COX-2, NF-*κ*B, PCNA, iNOS, VEGF, and disintegration of DNA	[170]
Thymoquinone and curcumin	Gentamicin-induced liver injury in rats	↓ deleterious effects on liver function and histological integrity	↑ antioxidant defense system; ↓ inflammation and apoptosis	[171]
Chrysin	Cisplatin-induced hepatic damage in rats	↓ hepatotoxicity	↓ oxidative stress and inflammatory response	[172]
Bazhen Decoction	Acetaminophen-induced acute liver injury in mice	↓ ALT, AST, ALP, LDH, TNF-*α*, IL-1*β*, ROS, TBARS, GSH depletion, and loss of MMP	↑ SOD, CAT, GR, and GPx; ↓ inflammatory mediators; ↓ Bax/Bcl-2 ratio and caspase-3, caspase-8, and caspase-9	[173]
Probiotic *Lactobacillus casei *Zhang	Endotoxin- and D-galactosamine-induced liver injury in rats	Antioxidative and anti-inflammatory effects	TLR4 signaling	[174]
Lutein	Guinea pigs fed a hypercholesterolemic diet	↓ oxidative stress and inflammation	↓ NF-*κ*B DNA binding activity	[175]
Galangin	Fructose-induced liver damage in rat	↓ oxidative damage	↓ inflammatory pathway	[176]
*Ganoderma applanatum *terpenes	Benzo(alpha)pyrene-induced liver damage in mouse	Decreased oxidative stress and inflammation	↑ antioxidant enzymes and suppressing inflammatory response	[177]
